# Intrauterine Malnutrition Reduced Long Leptin Receptor Isoform Expression and Proinflammatory Cytokine Production in Male Rat Pulmonary Endothelial Cells Stimulated by Lipopolysaccharide

**DOI:** 10.1155/2018/8597361

**Published:** 2018-07-09

**Authors:** Aleksandro M. Balbino, Marina M. Silva, Gabriela A. Azevedo, Noemi L. Gil, Renaide R. Ferreira, Leila A. dos Santos, Rebéca M. Gasparin, Liliam Fernandes, Maristella A. Landgraf, Richardt G. Landgraf

**Affiliations:** ^1^Department of Pharmaceuticals Sciences, Universidade Federal de São Paulo-Campus Diadema, Diadema, SP, Brazil; ^2^Biotério Central, Universidade de São Paulo, São Paulo, SP, Brazil; ^3^Department of Pharmacology, Universidade de São Paulo, São Paulo, SP, Brazil

## Abstract

**Background/Aims:**

We have previously shown that low birth weight (LBW) rats exposed to intrauterine malnutrition have an impaired lung inflammatory response and reduced levels of inflammatory mediators; however, circulating leptin levels were not increased. We evaluated long leptin receptor isoform (ObRb) expression in lung endothelial cells from low birth weight rats and examined its role in the production of lipid mediators and cytokines.

**Methods:**

Lung endothelial cells were obtained from normal birth weight (NBW) rats or LBW rats subjected to intrauterine malnutrition. These cells were stimulated with leptin (10 ng/mL), LPS (lipopolysaccharide, 1 *μ*g/mL), or leptin plus LPS. Six hours after stimulation, the production of inflammatory mediators (PGE_2_, LTB_4_, IL-1*β*, and IL-6) was evaluated using commercial ELISA kits, and Western blotting was performed to investigate p38MAPK, NF-*κ*B, and ObRb expression.

**Results:**

Leptin increased IL-1*β* levels in only cells from the NBW group, whereas LPS increased PGE_2_ and LTB_4_ levels in cells from both groups; leptin addition potentiated lipid mediator production induced by LPS in the NBW group. LPS enhanced the production of IL-1*β* and IL-6 in only endothelial cells from NBW rats. Leptin receptor expression was decreased (63%) in endothelial cells from LBW rats. None of the stimuli increased NF-*κ*B or p38 signaling pathway expression in cells from LBW rats.

**Conclusion:**

These results suggest that intrauterine malnutrition compromises leptin receptor expression and cytokine production in pulmonary endothelial cells stimulated by LPS; these effects seem to involve the NF-*κ*B and p38MAPK signaling pathways.

## 1. Introduction

Leptin (derived from the Greek word “leptos,” meaning thin) is a nonglycosylated peptide hormone with a molecular weight of 16 kDa; in murine animals, leptin is encoded by the obese gene (Ob), which shows 84% homology with the human leptin gene [1]. Leptin, primarily synthesized by adipocytes in white adipose tissue, is a pleiotropic molecule involved in promoting energy expenditure and satiety, as well as homeostasis, immunity, and reproductive neuroendocrine and neuroprotective functions [2–4]. These effects depend on binding to its receptor, long leptin receptor isoform (ObRb), a transmembrane protein with a cytoplasmic domain that activates signal transduction pathways, including the Janus kinase-signal transducer and activator of transcription (JAK-STAT) and mitogen-activated protein kinase (MAPK) signaling pathways [5, 6].

In a recent review, Kaczyńska et al. demonstrated the involvement of leptin in pulmonary physiology by stimulating the lung ventilation process and in different respiratory diseases; in asthma, leptin increases airway hyperresponsiveness and induces eosinophil accumulation, and in obstructive sleep apnea syndrome, leptin appears to be involved in the apnea-hypopnea index and the incidence of hypercapnia episodes [7]. Other authors have demonstrated that leptin negatively regulates LPS-induced lung injury and modulates corticosterone and insulin levels [8].

Acute malnutrition is associated with reduced leptin levels and immunosuppression [9, 10], cytokine expression deregulation [11], and decreased leukotriene synthesis in alveolar macrophages [12]. In a previous study, we observed that rats with a normal birth weight had increased circulating leptin levels after allergic stimuli in the lung, whereas rats with a low birth weight did not have increased circulating leptin levels or leukotriene production in the lungs after allergic stimulation; these results suggest that leptin plays a role in the reduced inflammatory responses of these animals [13].

The endothelium plays an important role in maintaining vascular tone and laminar blood flow and in cellular and molecular events related to immune reactions and responses to tissue injury [14, 15]. In vitro models of endothelial cells from lung tissue are important tools for understanding the interactions between the endothelium and leukocyte trafficking [16]. In lung tissue, a variety of cells, including bronchial epithelial cells, alveolar type II pneumocytes, macrophages, and mast cells, has been shown to produce leptin [17–19]; in addition, the lungs are the peripheral tissues with the highest ObRb expression levels [17, 20], and numerous cell types, including airway smooth muscle cells [21], bronchial and pulmonary epithelial cells [19], and endothelial cells [22], display high leptin receptor levels.

Proinflammatory cytokines, such as tumor necrosis factor-alpha (TNF-*α*) and interleukin-1*β* (IL-1*β*), stimulate the endothelium to express adhesion molecules (selectins, vascular cell adhesion protein 1 (VCAM-1), platelet endothelial cell adhesion molecule 1 (PECAM-1), and intercellular adhesion molecule-1 (ICAM-1)) that mediate the rolling, adhesion, and migration of leukocytes through the vessels [23].

Low birth weight rats exposed to intrauterine malnutrition have an impaired lung inflammatory response that is associated with reduced leukotriene B_4_ (LTB_4_), leukotriene C_4_ (LTC_4_) [11], and interleukin-6 (IL-6) [24] levels in the lung tissue; in addition, circulating leptin levels are not increased. Based on this information, we proposed that leptin might play an important role in the reduced production of inflammatory mediators in these animals. Therefore, we investigated whether leptin can modulate the production of inflammatory mediators in LPS-stimulated primary cultured lung endothelial cells from low birth weight rats exposed to intrauterine malnutrition. We also evaluated the participation of the NF-*κ*B and p38MAPK signaling pathways in this process.

## 2. Materials and Methods

### 2.1. Animals

Animal care and research protocols were in accordance with the principles and guidelines adopted by the Brazilian College of Animal Experimentation (COBEA), and this project was approved by the Ethical Committee for the Animal Research of the Federal University of São Paulo (CEUA 1038/11). Seven male and twenty female Wistar rats from the CEDEME colony (Federal University of São Paulo) were used to obtain offspring. The rats were housed in a 22 ± 1°C environment at 60% humidity and were maintained on a 12 h light-dark cycle. The male offspring of dams that were nourished and malnourished during the entire gestational period were used at 12 weeks of age. We avoided using female rats because of the fluctuation in their hormone levels (estrogen/progesterone). Males and females have different catabolism, which can significantly alter the regulation and production of several hormones [25]. These differences could modulate the inflammatory response and influence our results.

### 2.2. Protocol for the Induction of Intrauterine Undernutrition

Timed mating was carried out in age-matched (12 to 16 weeks old) female and male Wistar rats. To assess the estrus stage of the females, vaginal smears were checked before the males were introduced. Day 1 of the pregnancy was determined as the day when spermatozoa were detected in the vaginal smear. After confirmation that mating occurred, the rats were housed individually in standard rat cages. The female rats were divided randomly into two groups: nourished ad libitum and undernourished. The nourished female rats were fed with a standard commercial rat diet (Nuvital, Nuvital Nutrientes S/A, PR, Brazil) containing protein (minimum 22%), carbohydrates (maximum 54%), fat (minimum 4.5%), cellulose (maximum 8%), minerals (maximum 10%), water (maximum 12.5%), and vitamins. The undernourished female rats were fed with the same diet at 50% of the nourished female rat intake; this rate was determined according to the amount of food consumed by the control group from day 1 of the pregnancy until day 23 (parturition). All rats were fed daily in the morning, and consumption was determined 24 h later. After parturition, the dams received food ad libitum; therefore, the pups differed in only prenatal dietary experience. In a previous study, we demonstrated that the litter size and male to female ratio did not differ between the offspring from malnourished and nourished rats [26]. To prevent variation in neonatal growth due to the availability of milk during suckling, the litter sizes were standardized to eight pups on day 1. We did not observe a significant difference in the lung weight/birth weight ratio between the normal birth weight (NBW = 0.00597) and low birth weight (LBW = 0.00588) groups.

### 2.3. Endothelial Cell Isolation and Culture

Cell cultures were established according to procedures previously described by Chen et al. [27]. Randomly selected male rats from different litters were euthanized at 12 weeks of age by an overdose of anesthesia (ketamine/xylazine); then, their lungs were excised, washed with phosphate-buffered saline (PBS), cut into 1 × 1 × 1 mm^3^ pieces, and placed in six-well (35 mm) dishes. The tissues were covered with Dulbecco's modified Eagle's medium (DMEM-low glucose) supplemented with fetal bovine serum (FBS, 20%) and gentamicin (40 mg/L), pH 7.4 and placed in a CO_2_ incubator (Sheldon Mfg. Inc., USA) (37°C). Lung explants were discarded after 60 h, and the medium was changed every 2-3 days. The cells were grown to confluence and further propagated at a 1 : 4 ratio using trypsin (0.1%). The cells were maintained in culture until the fourth passage when all assays were performed.

### 2.4. Identification and Characterization of Endothelial Cells

Cells were seeded on sterile glass coverslips (13 mm) and fixed in paraformaldehyde (PFA, 4%) at room temperature for 30 min. The cells were permeabilized with Nonidet P40 (1%) and blocked with FBS (5%) in PBS for 30 min at 37°C. The samples were incubated overnight at 4°C with primary rabbit antibodies against von Willebrand factor (vWF) and *Ulex europaeus* lectin agglutinin I (UEA-1), which binds specifically to L-fucose residues on the endothelium (1 : 50 dilution). In all experiments, cellular staining was detected using bovine anti-rabbit IgG—Texas red-conjugated or goat anti-mouse IgG—FITC-conjugated secondary antibody at 1 : 100 dilution for 2 h at 37°C. Controls were obtained using coverslips incubated with only FBS, followed by secondary antibody. Cell nuclei were counterstained with 4,6-diamidino-2-phenylindole (DAPI) at 1 : 400 dilution for 5 min at 37°C. The coverslips were observed, and images were obtained by a fluorescence microscope (Axiovert 100M, Carl Zeiss SMT, Germany).

### 2.5. Inflammatory Stimulus (In Vitro)

Cells were seeded in six-well dishes (500,000 cells/well), and semiconfluent cultures were incubated with DMEM supplemented with fetal bovine serum for 24 h in an incubator (37°C/5% CO_2_). After 24 h, the cells were washed with ice-cold PBS; DMEM was added, and the cells were stimulated with LPS (1 *μ*g/mL) [28–30] and/or leptin (10 ng/mL) [31, 32] for 6 h. Then, the cells were collected in cold PBS and centrifuged at 1500 rpm for 5 min at 4°C. For the negative control group, only DMEM was added to the cells. Finally, the cells and the supernatant from the cells were collected and stored in a freezer at −80°C for all subsequent analyses.

### 2.6. Quantification of Lipid Mediators in the Cell Supernatant

PGE_2_ and LTB_4_ concentrations were determined by using EIA kits (Cayman Chemical Co., MI, USA) according to the method of Pradelles et al. [33]. The sensitivity of the LTB_4_ assay was 4.0 pg/mL, and that of the PGE_2_ assay was 15 pg/mL. The CV% values were as follows: LTB_4_: intra-assay < 8.37%, interassay < 24.41%; PGE_2_: intra-assay < 3.7%, interassay < 20.9%.

### 2.7. ObRb Receptor, NF-*κ*B p65, and Phospho-p38 MAPK Quantification by Western Blotting

Cells were stimulated with LPS (1 *μ*g/mL) and/or leptin (10 ng/mL) for 6 h; then, they were collected in cold PBS and centrifuged at 1500 rpm for 5 min at 4°C. The cell pellet was suspended in lysis buffer (Tris-HCl 50 mmol/L, pH 7.4, NaCl 100 mmol/L, and NP40 0.5%) with a protease/phosphatase inhibitor (Halth TM, Thermo Scientific, USA). Protein concentrations were determined by a BCA protein assay kit (Thermo Scientific, USA). Equal amounts of protein (55 *μ*g) were separated with 10% sodium dodecyl sulfate-polyacrylamide gel electrophoresis (SDS-PAGE). The proteins in the gel were transferred onto nitrocellulose membranes (0.45 *μ*m) and blocked for 60 min with 5% (wt/vol) nonfat dry milk diluted in TTBS (Tris base 0.2 mmol/L, NaCl 1.4 mmol/L, and Tween 20 0.1%), pH 7.6. The membranes were incubated overnight with polyclonal antibodies against ObRb (sc-8325 (H-300) rabbit polyclonal IgG, Santa Cruz Biotechnology Inc., EUA), NF-*κ*B p65 (number 3987, Cell Signaling; rabbit polyclonal IgG Tech, USA), or phospho-p38 MAPK (number 9215, Cell Signaling; rabbit polyclonal IgG Tech, USA) at 1 : 1000 dilutions. The blots were washed with TTBS (3 × 5 min) and incubated with a secondary horseradish peroxidase- (HRP-) conjugated goat anti-rabbit antibody (number 70745, Cell Signaling Tech, USA) at a 1 : 2000 dilution for 60 min at room temperature. ObRb receptor, NF-*κ*B p65, and phospho-p38 MAPK expression was detected by chemiluminescence (GeneGnome System, Syngene, UK) and quantified by densitometry (Gene Tools Software, UK). *β*-Actin expression was used as an internal control (1 : 2000 dilution, number 4970, Cell Signaling).

### 2.8. Quantification of Cytokines in the Cell Supernatant

Milliplex® map Kit-Rat Cytokine chemokine magnetic bead panels (EMD Millipore Corporation, Darmstadt, Germany) were used to measure IL-1*β*, IL-6, and leptin levels in the cell supernatant. The kits were used according to the manufacturer's instructions (MAGPIX™, Luminex®, MiraiBio, Alameda, CA). The data were analyzed using xPONENT® software (MAGPIX, Luminex, MiraiBio, Alameda, CA). The standard curves ranged from 1.95 to 32,000 pg/mL. The lower limits of detection for each cytokine were as follows: IL-1*β* (2.0 pg/mL), IL-6 (0.6 pg/mL), and leptin (10 pg/mL). The CV% values were as follows: IL-1*β*: intra-assay < 15%, interassay < 20%; IL-6: intra-assay < 10%, interassay < 20%; and leptin: intra-assay < 20%, interassay < 25%.

### 2.9. Statistical Analysis

Statistical analyses were carried out using GraphPad Prism software (v.6; GraphPad Software, San Diego, CA, USA). The body weight gain is presented as the mean percentage, and the other results are presented as the means ± SEM. Statistical evaluations of the data were determined by two-way analysis of variance followed by the Tukey-Kramer multiple comparison test. Student's *t-*test was used when necessary. A *P* value lower than 0.05 was considered statistically significant.

## 3. Results

### 3.1. Characteristics of the Offspring

**Figure 1:**
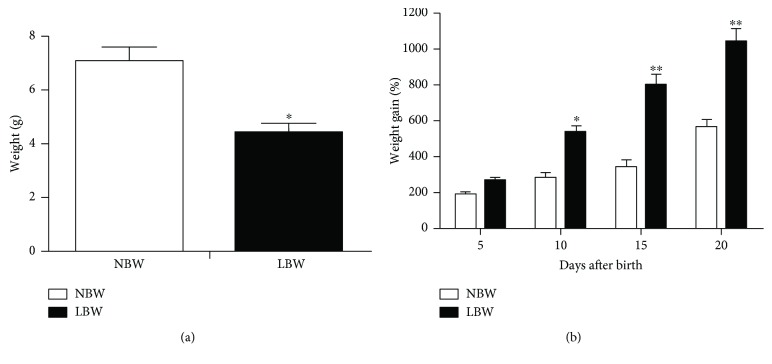
Offspring weight. (a) Offspring weight on the day of birth. The results are presented as the means ± SEM of 12–15 animals/group, ^∗^*P* < 0.05 compared to the NBW group. (b) Mean percentage of weight gain from day 0 until day 20. The results are presented as the mean percentage of weight gain ± SEM of 12–15 animals/group, ^∗^*P* < 0.05 and ^∗∗^*P* < 0.01 compared to the NBW group.

Litter size did not differ between the NBW and LBW groups, indicating that food restriction during the gestation period did not affect reproductive ability. These data agree with the results found by Landgraf et al. [26, 34]. The offspring from the undernourished dams throughout gestation had significantly lower birth weights than the nourished offspring (Figure 1(a)). After 10 days, the low birth weight (LBW) offspring had higher percentages of body weight gain than the normal birth weight offspring (NBW), and this difference remained until the twentieth day (Figure 1(b)).

### 3.2. Primary Cultured Endothelial Cells

**Figure 2:**
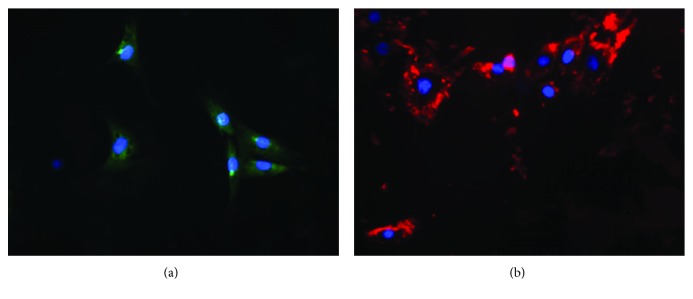
Immunostaining of specific endothelial cell markers. Staining for (a) *Ulex europeaus* lectin agglutinin I (UEA-1), green, and (b) von Willebrand factor (vWF), red. The nuclei were counterstained with DAPI solution for cellular localization. 400-fold increase.

Primary cultured endothelial cells obtained from the lung explants grew in a monolayer of polygonal cells, exhibited strong contact inhibition, and were characterized morphologically by a cobblestone appearance similar to that observed in a previous study [35]. Positive staining for UEA-1 (Figure 2(a)) and vWF (Figure 2(b)) was detected in more than 90% of the cells in culture.

### 3.3. Leptin Potentiated the LPS-Induced Secretion of Lipid Mediators in Only the Lung Endothelial Cell Supernatants from NBW Rats

**Figure 3:**
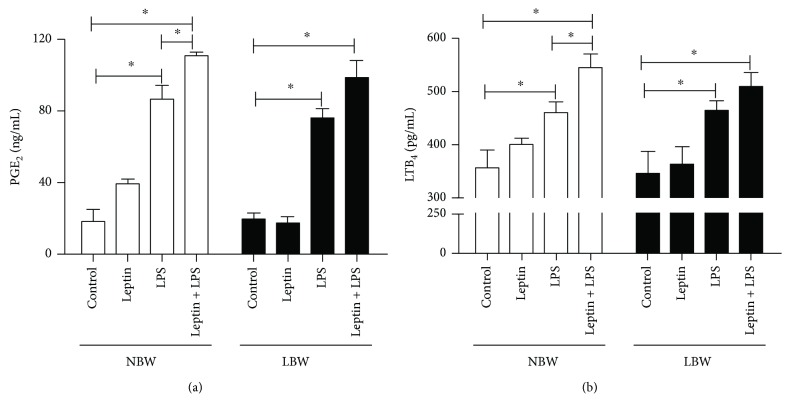
Effect of leptin on LPS-induced PGE_2_ and LTB_4_ secretion into the supernatants of pulmonary endothelial cells. PGE_2_ and LTB_4_ were measured in the supernatants of pulmonary endothelial cells using an EIA kit 6 h after stimulus with LPS and/or leptin. Cells were obtained from 8 male Wistar rats selected randomly from 6 different litters per group. The results are presented as the means ± SEM, ^∗^*P* < 0.05.

Two-way ANOVA showed no significant interaction effect of intrauterine growth restriction and treatment on PGE_2_ (*P* = 0.26) and LTB_4_ secretion (*P* = 0.86), but there was a significant main effect for treatment (*P* < 0.001). A significant increase in LPS-induced PGE_2_ and LTB_4_ secretion in lung endothelial cells cultured from both NBW and LBW was observed. The addition of leptin potentiated the LPS-induced production of PGE_2_ (86.33 ± 7.8 to 110.7 ± 2.1 ng/mL) and LTB_4_ (460.8 ± 19.9 to 545.0 ± 25.5 pg/mL) in the supernatants of lung endothelial cells from NBW rats but not from LBW rats (Figures 3(a) and 3(b)).

### 3.4. LPS Induced Cytokine Secretion from Lung Endothelial Cells from Only NBW Rats

**Figure 4:**
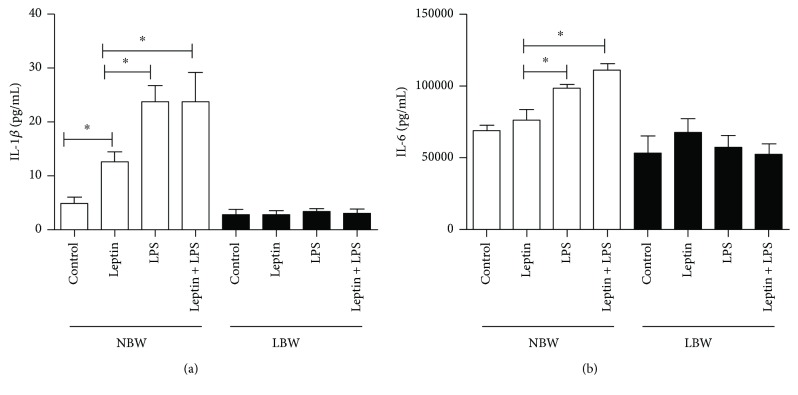
Effect of leptin on LPS-induced cytokine secretion into the supernatants of pulmonary endothelial cells. (a) Interleukin-1*β* (IL-1*β*) and (b) interleukin-6 (IL-6) were quantified in the supernatants of pulmonary endothelial cells 6 h after stimulus with LPS and/or leptin by multiplex assays as described in Materials and Methods. Cells were obtained from 8 male Wistar rats selected randomly from 6 different litters per group. The results are presented as the means ± SEM, ^∗^*P* < 0.05.

Lung endothelial cells from NBW rats showed increased IL-1*β* and IL-6 production after LPS stimulation. The addition of leptin did not alter the production of these cytokines in cells from NBW rats (Figures 4(a) and 4(b)). Lung endothelial cells from LBW rats produced neither IL-1*β* nor IL-6 leptin after stimulation. A significant interaction effect of intrauterine growth restriction and treatment on IL-1*β* (*P* = 0.002) and IL-6 (*P* = 0.02) production was confirmed by two-way ANOVA, indicating that these factors acted dependently. Moreover, the effects of intrauterine growth restriction (*P* < 0.001) and treatment (*P* < 0.001) on IL-1*β* secretion were also significant. Although two-way ANOVA revealed a significant effect of intrauterine growth restriction on IL-6 production, no significant effect was observed for treatment (*P* = 0.10).

### 3.5. Leptin Secretion from Lung Endothelial Cells

**Figure 5:**
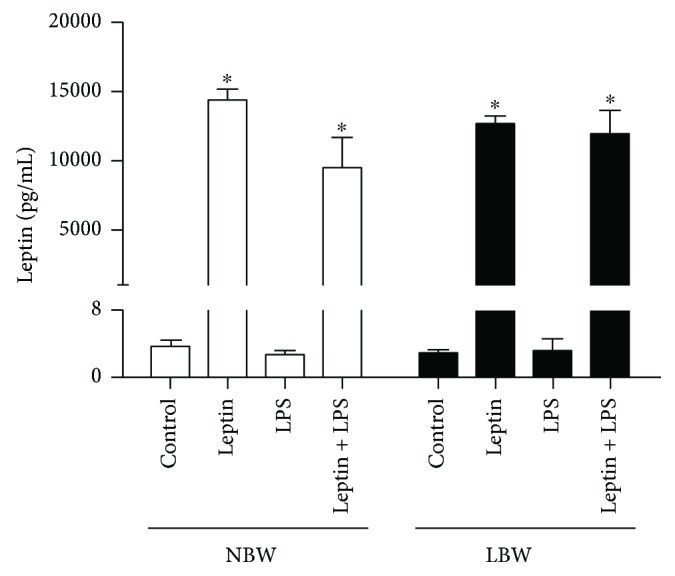
Effect of leptin on LPS-induced leptin secretion into the supernatants of pulmonary endothelial cells. Leptin was quantified in the supernatants of pulmonary endothelial cells 6 h after stimulus with LPS and/or leptin by multiplex assays as described in Materials and Methods. Cells were obtained from 8 male Wistar rats selected randomly from 6 different litters per group. The results are presented as the means ± SEM, ^∗^*P* < 0.05.

We observed increases in leptin levels after treatment with only leptin and leptin plus LPS (Figure 5), and this effect was confirmed by two-way ANOVA, which indicated a significant effect of treatment on leptin levels (*P* < 0.001).

### 3.6. Lung Endothelial Cells from LBW Rats Showed Decreased ObRb Receptor Expression

**Figure 6:**
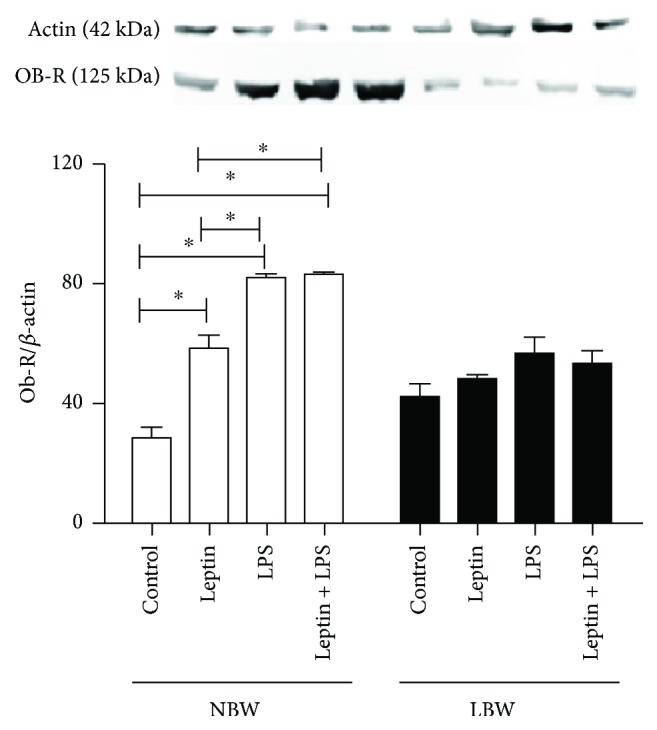
Effect of leptin on LPS-induced Ob-R expression in pulmonary endothelial cells. Endothelial cells were harvested 6 h after stimulus with LPS and/or leptin to quantify the expression levels of Ob-R using Western blotting. The graphs represent the band intensities determined by densitometric analyses and normalized to the total amount of *β*-actin present in each lane. Cells were obtained from 8 male Wistar rats selected randomly from 6 different litters per group. The results are presented as the means ± SEM, ^∗^*P* < 0.05.

In cells from NBW rats, both leptin and LPS enhanced ObRb expression, and LPS induced a significantly higher increase in ObRb expression than leptin; in addition, leptin did not potentiate the ObRb expression induced by LPS (Figure 6). ObRb receptor expression did not differ between the two groups under basal conditions, and the addition of leptin, LPS, or leptin plus LPS did not increase ObRb receptor expression in LBW rats (Figure 6).

### 3.7. Lung Endothelial Cells from LBW Rats Showed Decreased p38 MAPK and NF-*κ*B Pathway Expression

**Figure 7:**
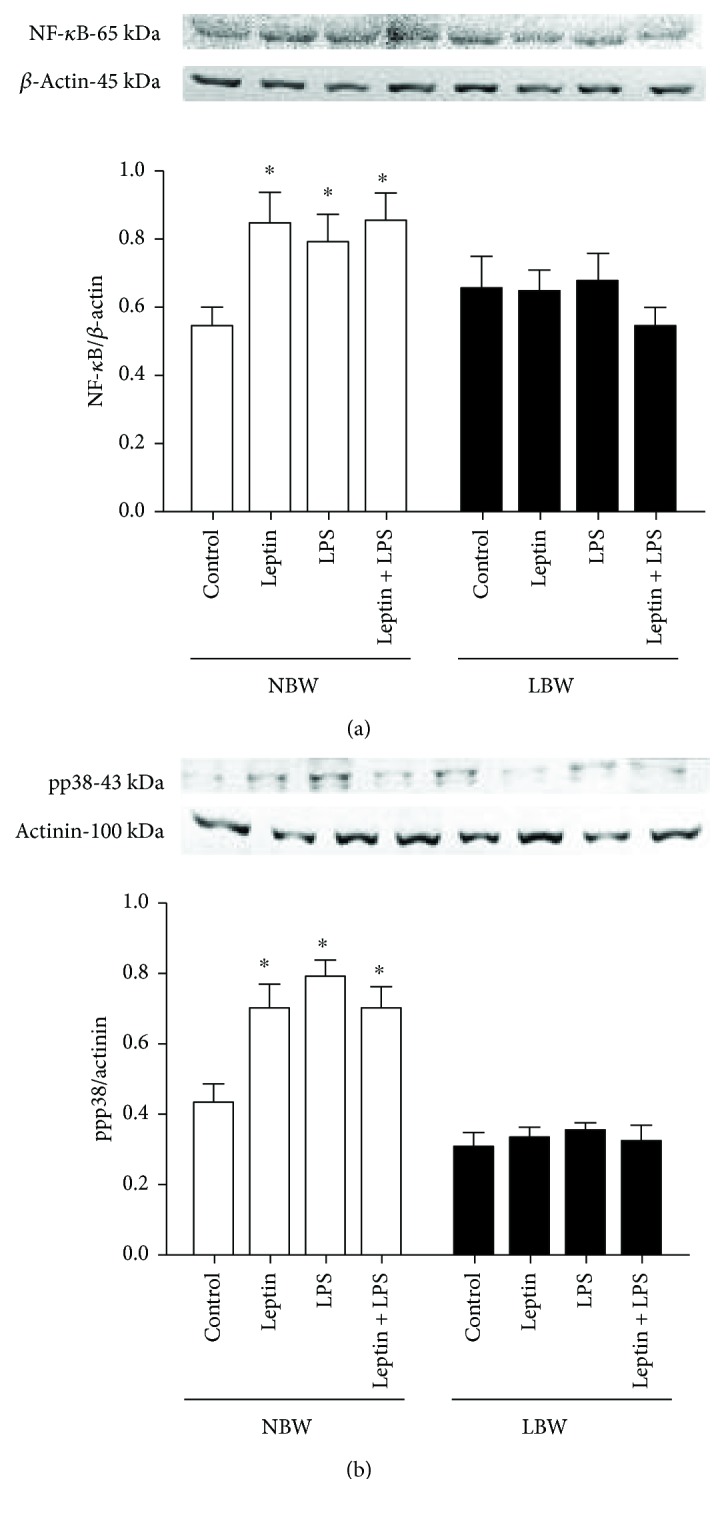
Involvement of the p38 kinase and NF-*κ*B pathways in the regulation of Ob-R expression on LPS-induced Ob-R expression in pulmonary endothelial cells. Endothelial cells were harvested 6 h after stimulus with LPS and/or leptin to quantify the activation of the p38 MAPK and NF-*κ*B pathways using Western blotting. The graphs represent the band intensities determined by densitometric analyses and normalized to the total amount of *β*-actin (NF-*κ*B) and *β*-actinin (pp38) present in each lane. Cells were obtained from 8 male Wistar rats selected randomly from 5 different litters per group. The results are presented as the means ± SEM, ^∗^*P* < 0.05.

We evaluated the p38 MAPK and NF-*κ*B pathway expression in endothelial cells obtained from lung tissues. Both LPS and leptin administration increased the NF-*κ*B and p38 pathway expression in cells from NBW rats (Figures 7(a) and 7(b)); in addition, leptin did not increase the p38 MAPK and NF-*κ*B pathway expression induced by LPS. In cells from LBW rats, none of the stimuli mediated changes in the p38 MAPK and NF-*κ*B pathways (Figures 7(a) and 7(b)).

## 4. Discussion

In the present study, we demonstrated that lung endothelial cells from intrauterine undernourished rats with a low birth weight had deficient IL-1*β* and IL-6 production after inflammatory stimuli. This deficiency could be associated with the lack of ObRb receptor expression.

Maternal prenatal undernutrition could result in important alterations in offspring development [36, 37]. Consistent with previous studies, we observed that the offspring of prenatally undernourished mothers presented with a 30% reduction in birth weight, followed by accelerated growth characterized by rapid weight gain [22]. Frisancho [38] suggested that this phenomenon might be a compensatory mechanism associated with reduced fat oxidation and increased carbohydrate metabolism.

In this study, we used a technique described by Chen et al. [27] and Loiola et al. [35]; lung endothelial cells derived from lung tissue explants were grown in FBS- (fetal bovine serum-) enriched medium, and no substances that could damage the structure and function of the pulmonary endothelial cells were added. Here, the lung endothelial cells were characterized using selective markers for these cells, namely, ULEX, which binds specifically to endothelial cell-specific glycoproteins and glycolipids [39], and the von Willebrand factor, which is a glycoprotein produced exclusively by endothelial cells and platelets [40].

Leptin is an adipocyte-derived protein encoded by the ob gene; although its plasma levels are directly correlated with body fat mass, this protein can be synthesized to a lesser extent by other tissues, such as brown adipose tissue, placenta [41], liver, stomach, intestine [42], hypothalamus, pituitary [43], skeletal muscle [44], and immune cells [45]. Leptin can modulate not only the endocrine system but also homeostasis, hematopoiesis, energy metabolism, innate and adaptive immune responses, and endothelial cell activation [31, 46].

Lipopolysaccharide (LPS), also termed endotoxin, is the major component of the outer membrane of Gram-negative bacteria [47]. It is considered a potent inducer of inflammation, and it directly activates the vascular endothelium, leading to the production of cytokines and inflammatory mediators and the expression of adhesion molecules, which contribute to diapedesis [48]. In the present study, LPS induced the production of lipid mediators and cytokines in endothelial cells from NBW rats, thus confirming the data from previous studies.

According to Rola-Pleszczynski and Stankova [49], two classes of soluble mediators act as important agents in the orchestration of the inflammatory response: lipid mediators and cytokines, which are synthesized from phagocytes and parenchymal cells. A loop involving eicosanoids and cytokine production has been previously demonstrated [49, 50]. The release of PGE_2_ and LTB_4_ augments the response to a variety of inflammatory stimuli, such as IL-1, IL-6, and TNF-*α*; on the other hand, leukotrienes could also modulate the release of these cytokines. However, in studying eicosanoid metabolism in porcine endothelial cells, Bustus et al. [51] demonstrated that the addition of anti-IL-1*β* antibodies did not alter cyclooxygenase-2 (COX-2) expression in stimulated endothelial cells, indicating that IL-1*β* was not responsible for the increases in COX-2 and PGE_2_ in that system. Our data demonstrated that the LPS stimulation of endothelial cells from LBW rats increased PGE_2_ and LTB_4_ but not IL-1*β* levels, indicating that the production of lipid mediators was not dependent on IL-1*β* in our model; these results contrast those observed by Bustus et al. [51].

The production of IL-1*β* during cell injury, infection, or inflammation occurs primarily in monocytes and macrophages, in addition to nonimmune cells, such as fibroblasts and endothelial cells [52]. In the present study, we found an important interaction effect of treatment with LPS and leptin and intrauterine growth restriction (and consequently low birth weight) on cytokine secretion. Leptin significantly increased IL-1*β* production and potentiated IL-1*β* production induced by LPS in cells from NBW rats, but the same effects were not observed in endothelial cells from LBW rats; regardless of the stimulus, IL-1*β* production remained at basal levels and did not change. Studies concerning the role of leptin in the production of inflammatory mediators are conflicting. It has been suggested that leptin has anti-inflammatory effects and that the administration of high doses of leptin could result in neutrophil inhibition in the lungs of rats with acute lung injury induced by acute pancreatitis [53]. Recently, we demonstrated that leptin downregulates LPS-induced acute lung injury by modulating corticosterone and insulin levels [8]. However, it has already been shown that leptin potentiates the effects of LPS on proliferation, monocyte activation, and proinflammatory cytokine production *in vitro* [54–56]. IL-1*β* and IL-6 stimulate the expression of adhesion molecules on the vascular endothelium and contribute to leukocyte migration [57]. It has already been demonstrated that LBW rats have reduced expression levels of adhesion molecules, such as L-selectin, P-selectin, and ICAM-1; these reductions can attenuate leukocyte migration [26]. Based on these data, we suggest that the failure of endothelial cells from LBW rats to produce IL-1*β* and IL-6 after LPS stimulation could be an important factor associated with reduced adhesion molecule expression and the consequently decreased leukocyte migration.

In endothelial cells, leptin receptor activation is associated with oxidative stress, chemokine and cytokine production, and adhesion molecule expression [57]. Mice lacking leptin receptors (db/db) are obese and present with a series of dysfunctions, such as hyperinsulinemia, increased cortisol levels, and impaired immune function [58, 59]. Leptin acts on target cells through interacting with its receptor, Ob-R, which is widely expressed in different parts of the body [60]. The expression of short (ObRa) and long (ObRb) leptin receptors has already been demonstrated in bronchial and alveolar epithelial cells in the lung [19, 61]. The ability of the short leptin isoform to activate the JAK signaling pathway is low, and it cannot activate the STAT pathway at all [62]. In addition, it has been demonstrated in adipocyte culture that the activating effects of leptin on the STAT3 and MAPK pathways are exerted through its long receptor [63]. Another study demonstrated that pulmonary macrophages express high levels of the long form leptin receptor; this isoform is the only one capable of inducing STAT3 signaling and is one of the main targets of leptin action in the lung [64]. Our results demonstrated that lung endothelial cells could not produce leptin after LPS stimulation. Both leptin and LPS additions significantly increased the expression of ObRb in endothelial cells from NBW rats, unlike what was observed in cells from LBW rats. Considering these data, we suggest that the reduced Ob-R response to the inflammatory stimuli might compromise the production of cytokines and lipid mediators in endothelial cells from LBW rats.

LPS can initiate several intracellular signaling events, such as stimulating pathways to activate NF-*κ*B and p38 MAPK, inducing cytokine expression [65, 66], and reducing proinflammatory cytokine expression, including IL-1*β* and TNF-*α*, to decrease NF-*κ*B and p38 MAPK pathway activation [67]. In addition, HUVEC exposure to leptin upregulates ObRb receptor expression and enhances NF-*κ*B activation and proinflammatory cytokine secretion [68]. Our data agree well with the literature data because we demonstrated that LPS and leptin-treated endothelial cells from NBW rats presented with increased proinflammatory cytokine expression and NF-*κ*B and p38 MAPK activation. In endothelial cells from LBW rats, increases in proinflammatory cytokine expression and NF-*κ*B and p38 MAPK activation were not observed, even after stimulation with LPS and/or leptin, suggesting that these pathways may be compromised in these animals and thus contribute to the decreased inflammatory response observed in our previous results [8, 13, 26].

In a previous study, our group demonstrated that LBW rats, despite normal basal leptin levels, did not have increased leptin levels after an inflammatory stimulus, and this inability to upregulate leptin levels was accompanied by an attenuated inflammatory response [13]. Here, the deficiency in ObRb expression likely reflects the ability of endothelial cells to adequately respond to LPS and leptin itself and contributes to the decreased production of inflammatory mediators.

Therefore, we suggest that the low birth weight induced by intrauterine malnutrition could induce molecular changes to the physiological responses of pulmonary endothelial cells to compromise the expression of ObRb and reduce the expression of inflammatory mediators and cytokines; our data also indicate the participation of the NF-*κ*B and p38 MAPK pathways in this process. These events could contribute to the attenuation of the inflammatory response observed in intrauterine malnourished rats with a low birth weight. Taken together, these data indicate a key role of leptin in the reduced inflammatory response of LBW rats.

## Data Availability

The data used to support the findings of this study are available from the corresponding author upon request.
